# Safety and Efficacy of Eculizumab Therapy in Multiple Sclerosis: A Case Series

**DOI:** 10.3390/brainsci11101341

**Published:** 2021-10-11

**Authors:** Marco Allinovi, Angelo Bellinvia, Francesco Pesce, Sabrina Milan Manani, Lorenzo Razzolini, Brigida Brezzi, Paolo Protopapa, Vittorio Mantero, Leonardo Caroti, Calogero Lino Cirami, Maria Pia Amato, Lucia Del Vecchio

**Affiliations:** 1 Nephrology, Dialysis and Transplantation Unit, Careggi University Hospital, 50139 Florence, Italy; carotile@aou-careggi.toscana.it (L.C.); ciramil@aou-careggi.toscana.it (C.L.C.); 2 NEUROFARBA Department, University of Florence, 50139 Florence, Italy; angelobellinvia@outlook.it (A.B.); dottlorenzo.razzolini@gmail.com (L.R.); mariapia.amato@unifi.it (M.P.A.); 3 Nephrology, Dialysis and Transplantation Unit, Department of Emergency and Organ Transplantation (DETO), University of Bari, 70121 Bari, Italy; f.pesce81@gmail.com (F.P.); paolo.protopapa.1990@gmail.com (P.P.); 4 Department of Nephrology, Dialysis and Transplant, San Bortolo Hospital, 36100 Vicenza, Italy; sabrina.milan@aulss8.veneto.it; 5 Division of Nephrology and Dialysis, SS Antonio e Biagio e C. Arrigo Hospital, 15121 Alessandria, Italy; bbrezzi@ospedale.al.it; 6 MS Center, Department of Neurology, ASST Lecco, 23900 Lecco, Italy; v.mantero@asst-lecco.it; 7 IRCCS Don Carlo Gnocchi, 50143 Florence, Italy; 8 Nephrology and Dialysis, ASST Lariana, 22100 Como, Italy; luciadelvecchio@yahoo.com

**Keywords:** multiple sclerosis, complement, eculizumab, interferon beta, disease-modifying therapy, adverse event, anticomplement therapy

## Abstract

(1) Background: Complement system activation has been proposed as one of the different factors that contribute to Multiple Sclerosis (MS) pathogenesis. In this study, we aimed to describe the potential effects of eculizumab, an anticomplement therapy, on MS disease activity in a cohort of relapsing–remitting (RR) MS patients who discontinued IFN-β therapy due to IFN-β-related thrombotic microangiopathy (TMA) onset. (2) Methods: In this retrospective observational multicentric study, we searched for all patients with MS treated by eculizumab with a survey of several nephrological and neurological centers (over 45 centers). (3) Results: Nine patients were included. The mean follow-up time under eculizumab was 3.72 ± 2.58 years. There were no significant differences in disease activity (EDSS, relapses, new T2, and/or Gd-enhancing lesions at MRI) considering the two years before and after eculizumab therapy. No adverse events potentially related to eculizumab therapy were reported during follow-up. (4) Conclusions: In this preliminary study, we described a good safety profile for eculizumab therapy in MS. However, the available data are not sufficient to make firm conclusions about the possible efficacy of eculizumab as a disease-modifying therapy for MS patients.

## 1. Introduction

Multiple Sclerosis (MS) is an inflammatory and neurodegenerative disease of the central nervous system (CNS) with a multifactorial etiology [1,2]. Complement system dysregulation has been proposed as one of the different factors that could contribute to MS pathogenesis and clinical manifestations [3]. The complement system is a critical innate immune defense against infection and an important driver of inflammation; however, these properties can also be harmful. Indeed, the complement system seems to have both a detrimental and a protective role on MS pathogenesis and the disease course [4]. In particular, several studies have suggested that complement overactivation may contribute to the worsening pathology that underlies MS progression [5,6,7,8,9]. Moreover, preclinical and clinical studies in the last decade have showed that the complement system has an established role in active plaque development [3]. Immunoglobulins, C1q and C5b-9 are present in all plaques, suggesting a dominant role for the classical pathway (triggered by immune complexes) in the demyelination process [10]. That given, complement components can be used as biomarkers of disease state activity and response to treatment in MS patients [10], and drugs targeting complement activity might be potentially helpful in MS therapy [11]. Interferon-β (IFN-β) is one of the most-used disease-modifying therapies (DMTs) in relapsing–remitting (RR) MS. IFN-β has also been proven safe and effective in a long-term follow-up, and the collateral effects are usually mild and often transitory. However, in recent years, growing evidence about a relationship between the use of IFN-β and thrombotic microangiopathy (TMA) development has been described through several case series [12,13,14,15]. TMA consists of the triad of consumptive thrombocytopenia, microangiopathic hemolytic anemia, and signs of ischemic damage in different organs (and, particularly, acute kidney injury). Among different forms of TMA, thrombotic thrombocytopenic purpura (TTP) is characterized by deficient ADAMTS-13 activity, with atypical hemolytic uremic syndrome (aHUS) by the dysregulation of the complement system’s alternative pathway [16]. Depending on the underlying etiology, therapy is usually based on plasma exchange, supportive therapies, the withdrawal of causative agents, and anticomplement therapy. Eculizumab (Soliris™, Alexion Pharmaceuticals, New Haven, CT, USA), a terminal complement inhibitor, is a humanized monoclonal antibody that binds with high affinity to the human C5 complement protein and blocks the generation of proinflammatory C5a and C5b-9. Eculizumab is approved for the treatment of Paroxysmal Nocturnal Hemoglobinuria and aHUS [17], but its use is also widely accepted for patients with severe secondary TMAs [18,19]. Anticomplement therapies may play a central role in treating CNS inflammatory and degenerative disorders [11,20]. Two recent articles have provided evidence for the long-term safety and sustained efficacy of eculizumab for refractory generalized myasthenia gravis [21] and Neuromyelitis Optica Spectrum Disorder (NMOSD) [22], leading to a renaissance of complement research in neuroscience. These studies’ results might suggest a potential role of eculizumab as a therapeutic option in other neuroinflammatory disorders of the CNS, such as MS. Although the complement comprises a cluster of activation pathways triggered in various ways and progressed through several different pathogenetic mechanisms in MS, specific treatment with agents that inhibit complement activation may interfere with the mechanisms involved in the pathogenesis of neurological disability in patients with MS. We hereby report the first study on a small group of RRMS patients treated with eculizumab after developing TMA due to IFN-β therapy. We aimed to describe the potential effects of eculizumab treatment on the MS disease activity in a small sample of RRMS patients who had to stop treatment with DMTs for TMA onset.

## 2. Materials and Methods

In this retrospective observational multicentric study, we searched all patients with MS treated by eculizumab because they developed TMA during IFN-β treatment with a survey of several nephrological and neurological centers (over 45 centers) in Italy, France, and the United States. All of the included patients were consecutively referred to their respective departments from 2011 to 2018. The included patients’ clinical data were extracted from the archives of the neurological department of each center, where they were recorded at the first visit and every six months or at relapses.

Exclusion criteria: Coexisting therapy with eculizumab and DMTs, eculizumab therapy <12 months, and unavailable data during the neurological follow-up (in terms of EDSS and MRI activity at different time points).

This study was conducted according to the Declaration of Helsinki principles, and informed written consent was obtained from each subject or each subject’s guardian at enrollment.

To evaluate eculizumab’s treatment efficacy, we used the recorded EDSS values two years before and after the eculizumab treatment. As for radiological disease activity, MRI activity was defined as new/enlarging T2 lesions or new Gadolinium-enhancing lesions. The transition to SPMS was defined according to the neurologist definition [23,24].

Eculizumab was prescribed at a fixed dosing schedule: a weekly injection of 900 mg eculizumab during the first 4 weeks of therapy after TMA onset, followed by a dose of 1200 mg in the fifth week. This higher dose is subsequently continued as maintenance therapy every 2 or 3 weeks. Complement activity was also wholly suppressed in all the patients when the infusions were spaced out at 3-week intervals.

Since patients treated with eculizumab are at a >1000 fold increased risk of meningococcal disease, the patients were vaccinated with meningococcal serogroup B and serogroup A, C, W, and Y vaccines.

Six out of the nine cases have been described in previous articles from a nephrological [13,14,25] or a neurological [26] point of view, not analyzing the potential role of eculizumab in MS, and with a much shorter follow-up.

The data were analyzed with SPSS software version 20.0 (SPSS Inc., Chicago, IL, USA). The demographic and clinical characteristics were described as the frequency (percentage) and mean ± standard deviation (SD). Intra-group comparisons were assessed through the McNemar test for related samples of the categorical variables or the Wilcoxon signed-rank test for continuous variables to compare the disease activity in the two years before and after the eculizumab treatment. We then analyzed the EDSS longitudinal trajectory over time by using an ANOVA test for repeated measures.

## 3. Results

Fourteen patients were screened for inclusion. Nine patients were included. Five patients were excluded for an eculizumab therapy duration <12 months or concomitant treatment with eculizumab and any DMT (fingolimod), respectively. According to the inclusion and exclusion criteria, all the included patients had been treated in Italian hospitals. The main demographical and clinical characteristics of the whole sample are depicted in Table 1.

**Figure 1:**
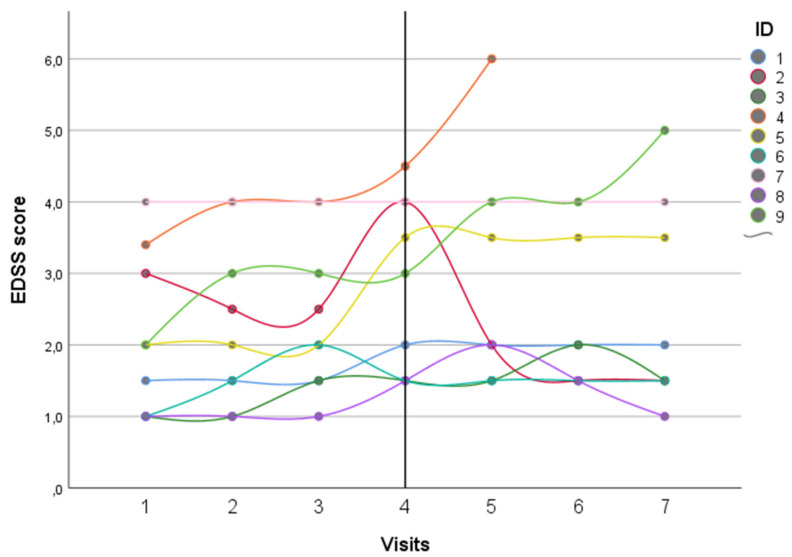
Temporal trends of the EDSS score for each patient during the two years before and after eculizumab therapy. Legend: EDSS = Expanded disability status scale and ID = Identification. Black vertical line represents the TMA onset, characterized by IFN-β withdrawal and eculizumab start.

All the patients had an RRMS phenotype and a mean age at onset of 24.2 ± 7.03 years, with relatively mild disability (median EDSS score 2, IQR 1.5–4.5). All of them were treated with IFN-β, two with Βferon™ and the other seven patients with Rebif™. Among these, four were previously treated with Avonex™ and switched to Rebif™ due to poor tolerance. All our patients received IFN-β >65 μg per week (seven out of nine patients >120 μg per week) for 13.9 (range 7–19.5) years. The longitudinal EDSS trajectories for each patient are depicted in Figure 1.

The mean age at inclusion was 40.7 ± 7.53 years, with a mean MS disease duration of 16.8 ± 2.9 years. One patient experienced mild clinical activity, and four had radiological disease activity in the year before TMA onset, with one patient who also had a Gd-enhancing lesion (Table 2).

Among the eight patients who tested the serum complement factors, five patients showed low C3 levels. Moreover, among the seven patients who performed a genetic analysis, three were negative and one had a causative MCD mutation (c.1058C > T) and a heterozygous CFHR3/CFHR1 gene deletion, while three had a predisposing genetic variant to aHUS: one patient with a heterozygous CFHR3/CFHR1 gene deletion, one patient was homozygous for the risk haplotype CFH-H3 and presented a variant of unknown significance (c.2650T > C) in the CFH gene, and one patient was heterozygous for both CFHR3/CFHR1 gene deletion and haplotype CFH-H3.

After treating the disorder’s acute phase, all the included patients suspended IFN-β therapy and initiated treatment with eculizumab. None of them were switched to another DMT.

**Figure 2:**
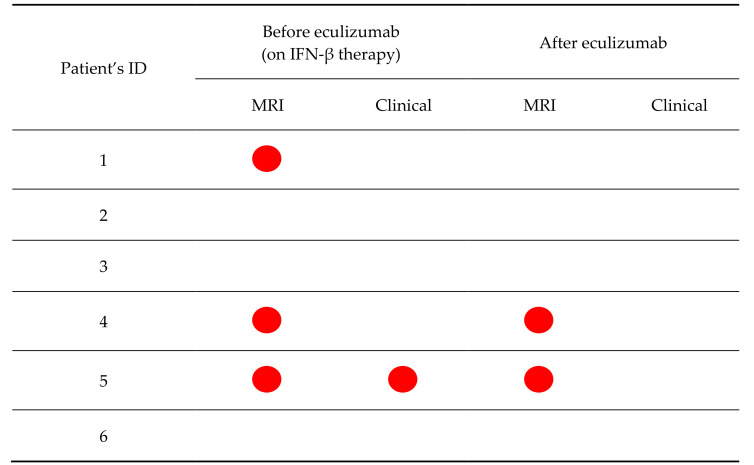

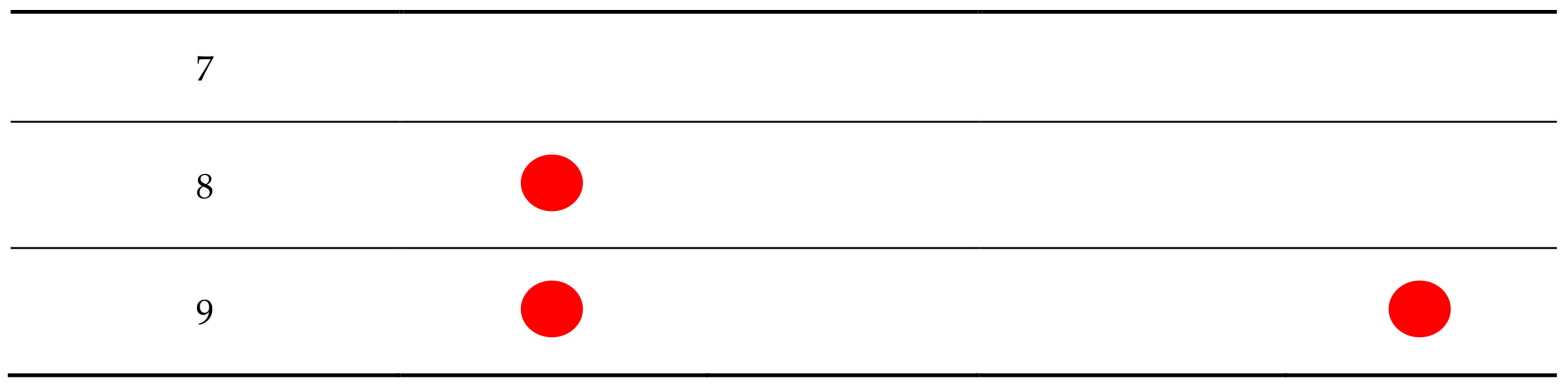
Radiological and clinical activity in the two years before and after eculizumab treatment. Red dots represent the clinical and radiological activity before and after starting the treatment with eculizumab. Legend: MRI = Magnetic Resonance Imaging.

Two patients experienced a relapse during eculizumab treatment after one and three years from IFN-β suspension, respectively (Figure 1 and Figure 2). The relapses were mildly disabling and completely resolved after a short corticosteroid treatment. Two patients experienced isolated radiological activity, with new T2 lesions detected during follow-up, all within two years from starting eculizumab therapy (Figure 2). For three patients, contrast-enhancement data were not available at the follow-up MRIs, because Gd was not administered for severe chronic kidney injury. None of the other six patients who had a contrast-enhanced MRI had Gd^+^ lesions at their follow-up MRIs.

At the last neurological visit, the mean EDSS score was stable for the whole cohort (median EDSS score 3, IQR 1.5–4.0), with no patients experiencing a significant disability worsening after a mean treatment duration of 3.7 ± 2.6 years. Interestingly, one patient with the active disease showed a significant EDSS score improvement with eculizumab therapy, even better than the EDSS score reached with the previous IFN-β therapy. No patients experienced a transition to a secondary progressive disease course. There were no significant differences in disease activity (EDSS, relapses, new T2, and/or Gd-enhancing lesions at MRI) considering the two years before and after the eculizumab therapy (Table 2). The ANOVA test disclosed no significant differences in the EDSS scores recorded before and during the treatment with eculizumab (*p* = 0.317, not shown).

Four patients suspended eculizumab during follow-up, one after eight months, because of no documented renal improvement, one after 2 years and two after about 3 years, respectively, for complete TMA resolution. Three of the four abovementioned patients re-initiated DMT treatment with a subsequent disease stability. One patient is currently not under any DMT. The other five patients are still under eculizumab treatment.

As for safety issues, no serious adverse events potentially related to eculizumab therapy were reported during follow-up. Three patients (33%) experienced mild adverse events (headache, nausea, and fatigue, respectively) that did not make eculizumab withdrawal necessary.

Finally, as for the nephrological outcomes, three (33.3%) patients resolved the TMA without further complications, while six (66.7%) patients needed dialysis treatment at TMA onset, of whom four patients discontinued dialysis after a mean of 2 months.

## 4. Discussion

The complement system has an established role in the pathogenesis of MS. Complement activation and dysregulation can occur in MS, as it has been demonstrated in white (with a deposition of C1q, C3d, and C5b-9 on inflammatory cells, astrocytes, and in blood vessel walls) [3,27] and grey (where an association between C1q cell density and tissue lesions has been detected) [9] matter lesions in MS pathology and could be one of the drivers of inflammation and/or neurodegeneration. That given, anticomplement therapies might also be beneficial in MS as DMTs. Although this therapy has proved its efficacy in reducing relapses compared to a placebo in NMOSD patients [22], currently, there are no studies about treatments with eculizumab or other anticomplement therapies in MS.

This case series represents the largest sample of MS patients treated by anticomplement therapy. All patients discontinued their treatment with IFN-β without replacing it with other DMTs, so it was possible to study the role of eculizumab in preventing MS clinical and radiological activity. In the nine included patients, after the suspension of IFN-β and initiation of treatment with eculizumab, no breakthrough disease activity suggesting a rebound has been recorded shortly after IFN suspension, and the clinical and radiological activity did not differ between the two years before and after eculizumab initiation (Table 1, Figure 1 and Figure 2). Even if the small sample size and the study design do not permit to prove the efficacy of eculizumab as a DMT for MS, our data showed a good safety profile, without a clear rebound in disease activity after IFN-β suspension. On the other hand, the included patients were mildly disabled and on long-term IFN-β treatment, suggesting a more benign disease course and, therefore, lower probability of detecting disease activity after DMT withdrawal.

In general, eculizumab showed an optimal safety profile, and most of the treated patients experienced no major adverse events, with only mild infusion reactions, such as hypertension, tachycardia, hypotension, headache, insomnia, fatigue, dizziness, skin rash, pruritus, diarrhea, vomiting, nausea, and abdominal pain. The administration of meningococcal vaccines is recommended for patients receiving eculizumab before beginning treatment.

In our cohort, we did not report any severe adverse effect. One-third of the included patients experienced mild adverse effects, while no cases of meningococcal infection or infusion-related events were reported; these data are in line with the scientific literature, which shows an optimal safety profile for eculizumab [22].

All the patients were treated with IFN-β at the usual dosage administered in MS, albeit being under a higher cumulative IFN-β dosage than recommended in the nephrological guidelines. A high IFN-β dosage for weight might be suspected as a potential risk factor for TMA, especially in patients with a long-tolerated administration of doses exceeding 50 μg per week and a low body mass index [15,28].

The main strengths of our study are the long-term follow-up and probably the largest, albeit still small, sample of MS patients treated with eculizumab therapy that could be recruited worldwide nowadays. On the other hand, our study suffered from some limitations, such as its retrospective nature, the small sample size, and the lack of a control group.

## 5. Conclusions

In conclusion, our preliminary study showed a good safety profile for eculizumab therapy in MS patients. Although the absence of a rebound after IFN-β suspension can be considered encouraging, the data collected were not sufficient to make any firm conclusions about the possible efficacy of eculizumab as a DMT for MS. Future randomized controlled trials or carefully designed longitudinal observational studies are needed to better determine the safety and efficacy profile of eculizumab in patients with MS.

## Figures and Tables

**Table 1:** Clinical and demographical characteristics of the whole cohort.

	Patients (*n* = 9)
Females, *n* (%)	5 (55.6)
Age, mean, years (SD)	45.3 (5.3)
Age at MS onset, mean, years (SD)	24.2 (7)
Length of IFN-β therapy, mean, years (SD)	13.9 (3.9)
Length of follow-up on eculizumab therapy, mean, years (SD)	3.72 (2.58)

Legend: SD, Standard Deviation; EDSS, Expanded Disability Status Scale; IQR, Interquartile Range; MRI, Magnetic Resonance Imaging; MS, Multiple Sclerosis; TMA, Thrombotic Microangiopathy.

**Table 2:** Disease activity in the two years before and after eculizumab therapy.

	Pre-Eculizumab (*n* = 9)	Post-Eculizumab (*n* = 9)	*p*
Relapses, *n* (%)	1 (11.1)	1 (11.1)	0.999 ^3^
New MRI T2 lesions, *n* (%)	4 (45)	2 (22.2) ^1^	0.625 ^3^
New MRI Gd+ lesions, *n* (%)	1 (11.1)	0 (0.0) ^2^	0.999 ^3^
EDSS Score, median (IQR)	2 (1.5–4.5)	3 (1.5–4.0)	0.898 ^4^

Legend: SD, Standard Deviation; EDSS, Expanded Disability Status Scale; IQR, Interquartile Range; MRI, Magnetic Resonance Imaging. ^1^ = available for 8 patients; ^2^ = available for 6 patients; ^3^ = McNemar test for related samples; ^4^ = Wilcoxon signed-rank test.
